# Intrapelvic bulboprostatic repair after urethral distraction injury and failed bulbomembranous anastomosis

**DOI:** 10.1016/j.eucr.2020.101221

**Published:** 2020-04-22

**Authors:** Peter Rehder, Lukas Andrius Jelisejevas, Marco Pedrini, Alexandra Gulacsi, Wolfgang Horninger, Jannik Stuehmeier

**Affiliations:** Medical University Innsbruck, Department of Urology, 35 Anich Street, 6020, Innsbruck, Austria

**Keywords:** Bulboprostatic anastomosis, Intrasphincteric dissection, Urethral distraction injury, Urethroplasty

## Abstract

A failed end-to-end anastomosis after membranous urethral distraction injury post-trauma is a surgical challenge. We present a case of a young man after complex pelvic injury. Revision urethroplasty was done utilizing nerve and vessel sparing techniques. Intrasphincteric dissection enabled bulbous urethral pull-through with intrapelvic anastomosis with good success. Low dose tadalafil was given to optimize penile rehabilitation.

## Introduction

Urethral repair after complex pelvic injury is challenging. Fracture(s) of the pelvic rim and/or symphysis may cause a disruption of urethral continuity and displacement resulting in malalignment. Furthermore the sphincter apparatus and the bladder neck may be injured to various degrees. The principles of treatment are not to cause further damage by inadvertent transurethral catheterization, or inappropriate attempts to align the non-approximate urethral ends. It may be better to initially place a suprapubic catheter, rather than force some kind of railroading technique to drain the bladder. After pelvic fracture treatment, patient mobilization and appropriate wound healing, urethroplasty is planned. Definitive repair of the damaged membranous (=sphincteric) urethra involves alignment with end-to-end anastomosis, vessel sparing dissection and preservation of the rhabdosphincter and the dorsal neurovascular bundle. We describe a case of function preserving urethroplasty utilizing a combined perineal and suprapubic approach anastomosing the mobilized bulbous urethra to the severed prostatic urethra. What makes this case noticeable is the fact that definitive repair was performed after a previously failed attempt of posterior urethroplasty. A spatulated end-to-end anastomosis was done within the small pelvis tunnelling the bulbous urethra through the rhabdosphincter.

### Case presentation

**Fig. 1:**
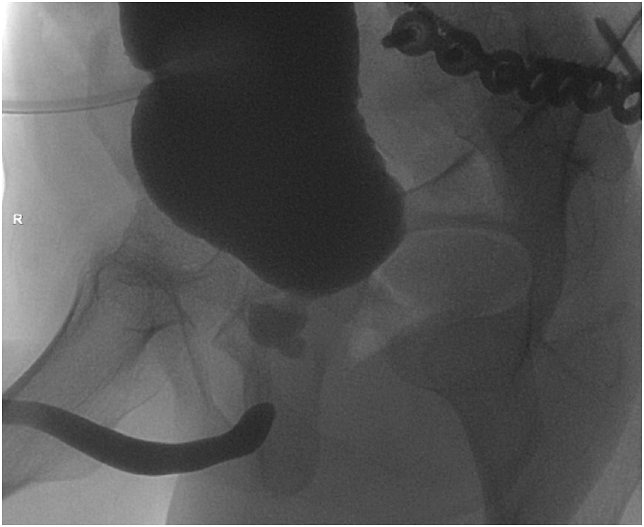
Combined cystogramm and retrograde urethrography showing total occlusion and malalignment between proximal prostatic urethra and membranobulbous urethra.

**Fig. 2:**
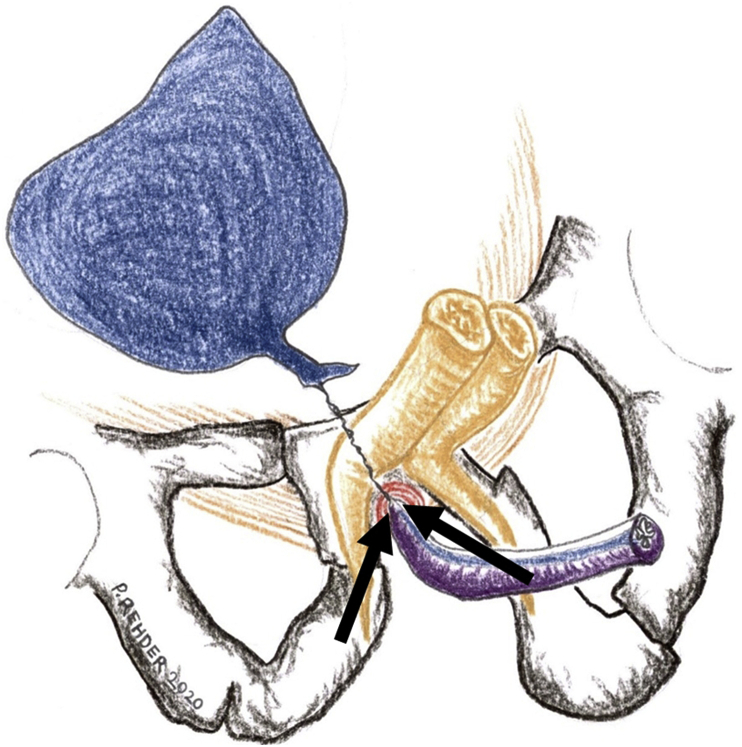
Arrows indicating plane for intrasphincteric dissection of rhabdosphincter. This allows for urethral pull through into small pelvis.

**Fig. 3:**
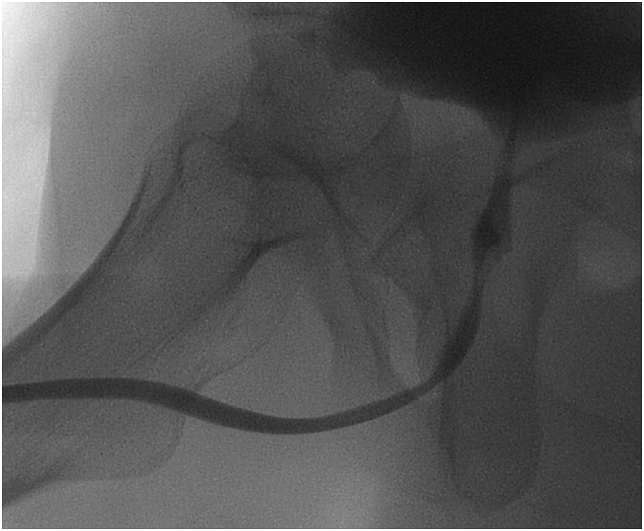
Micturating cystourethrogramm demonstrating open bladder neck, prostatic urethra, membranous urethra (relative narrowing) and unobstructed bulbous urethra six months after definitive repair.

A 20-year-old man suffered a severe crush injury of the pelvis with concomitant membranous urethral distraction injury. He underwent an attempt to posterior repair three months post-trauma using a perineal approach with bulbomembranous anastomosis. Inadvertently, the anastomosis was performed onto the posterior surface of the prostate. The prostatic apex was dislodged anteriorly (Fig. 1). As a result, the patient went into immediate retention after postoperative catheter removal. An anatomical midline dissection using a combined perineal and suprapubic approach was chosen six months after the previous attempt. Vessel and nerve sparing dissection was used preserving the rhabdosphincter. The bulbous urethra was completely mobilized via a midline perineal approach, excising all excess scar tissues. The retrograde blood supply of the urethral bulb via the dorsal penile vessels was still satisfactory. There was no visible connection to the proximal urethral end. The distal end of the membranous urethra can almost always be identified directly underneath the symphysis, just proximal to the divergence of the corpora cavernosa. Care should be taken not to damage the dorsal neurovascular bundle of the penis that runs behind a thin fascial sheet closer toward the symphysis.^1^ After identifying the rhabdosphincter intrasphincteric dissection is used.^2^ There is a fascial plane on the inner side of the rhabdosphincter allowing for lengthwise sliding of the urethral wall. This plane allows for careful dissection (Fig. 2). The mobilized bulbous urethra was now pulled through the rhabdosphincter. It was not possible to find the proximal urethral end within severe scar tissue. Through a lower midline laparotomy the bladder outlet and prostate was explored. Endoscopy through the site of the suprapubic catheter revealed that the prostatic apex was dislodged anteriorly right behind the symphysis pubis. Here the prostatic urethra was opened. No mobilization of the bladder or the remains of the prostate was possible above the pelvic floor. Through the perineal incision the proximal corpora cavernosa were dissected in the midline, again making sure not to damage the dorsal neurovascular bundle of the penis behind. This manoeuvre allows for the short cut needed to pull through the fully mobilized bulbopenile urethra into the small pelvis. A tension-free end-to-end anastomosis was now done intrapelvically very similar to the vesicourethral anastomosis after radical prostatectomy. A size 16 French transurethral catheter with longitudinal grooves was inserted. The patency of the anastomosis was checked by injecting saline solution alongside the catheter. No drain was used followed by layered wound closure. The transurethral catheter was left in situ for three weeks. Tadalafil 5mg per day was given to optimize penile rehabilitation for 6 months.^3^ Half a year after definitive repair the patient is acceptably continent using one safety pad per day (Fig. 3). Furthermore he has erections enough for masturbation and orgasm, although not enough for penetration.

## Discussion

Functional reconstruction after membranous urethral distraction injury and failed posterior urethroplasty seems possible in select cases.^4^ With care to detail to preserve the vessels, the dorsal neurovascular bundle, and the rhabdosphincter, we recommend another attempt to functional repair in these unfortunate patients. Correctly identifying the prostatic apex/proximal membranous urethral stump enables successful anastomosis. The literature supports giving tadalafil to prevent corporal fibrosis and veno-occlusive dysfunction in rats,^5^ although this has not yet been adequately described post-urethroplasty in humans.

## Conclusion

Revision surgery is possible after previous posterior urethroplasty with satisfactory functional outcome. Intrasphincteric preparation enables preservation of continence.

## Consent

Patient data anonymized and consent obtained for publication of data.

## Declaration of competing interest

None.

This research did not receive any specific grant from funding agencies in the public, commercial, or not-for-profit sectors.

## References

[1] Jordan G.H., Eltahawy E.A., Virasoro R. (2007). The technique of vessel sparing excision and primary anastomosis for proximal bulbous urethral reconstruction. J Urol.

[2] Gomez R.G., Scarberry K. (2018). Anatomy and techniques in posterior urethroplasty. Transl Androl Urol.

[3] Kim S., Sung G.T. (2018). Efficacy and safety of tadalafil 5 mg once daily for the treatment of erectile dysfunction after robot-assisted laparoscopic radical prostatectomy: a 2-year follow-up. Sex Med.

[4] Gupta N.P. (2008). Does a previous end-to-end urethroplasty alter the results of redo end-to-end urethroplasty in patients with traumatic posterior urethral strictures?. Int J Urol.

[5] Kovanecz I. (2008). Chronic daily tadalafil prevents the corporal fibrosis and veno-occlusive dysfunction that occurs after cavernosal nerve resection. BJU Int.

